# TOT Approach in stress urinary incontinence (SUI) – outcome in obese female

**DOI:** 10.1186/1471-2490-14-20

**Published:** 2014-02-20

**Authors:** Carsten Frohme, Friederike Ludt, Zoltan Varga, Peter J Olbert, Rainer Hofmann, Axel Hegele

**Affiliations:** 1 Department of Urology and Pediatric Urology, University hospital Marburg, Philipps University, Marburg, Germany; 2 Department of Urology, District hospital Sigmaringen, Sigmaringen, Germany

**Keywords:** Body mass index, Obesity, Obese female, Stress urinary incontinence, Transobturator tape (TOT)

## Abstract

**Background:**

Only limited data are available on the outcome of tension-free obturator tape (TOT) procedures in overweight and obese women. We would like to verify the objective and subjective outcomes of TOT in women with a higher body mass index (BMI).

**Methods:**

We evaluated the records of 116 patients who had undergone TOT, stratifying by BMI into normal weight (n = 31), overweight (n = 56), and obese (n = 29) groups. We compared pre- and postoperative evaluations, including subjective and objective outcome of TOT, complications, and quality of life assessed by validated questionnaires (ICIQ-SF and KHQ).

**Results:**

The median follow-up was 21 months. There were no significant differences between different groups in terms of objective cure rate and subjective success, quality of life scores and postoperative complications.

**Conclusions:**

Our data demonstrate that TOT procedure is safe and effective. BMI did not influence the outcome of TOT procedures at a median of 21 months after surgery and represents no contraindication for continence surgery. The success of the outcome of TOT procedure in females and the occurrence of complications are not negatively affected by obesity.

## Background

Stress urinary incontinence (SUI) is the complaint of involuntary loss of urine on effort or physical exertion (e.g. sporting activities), or on sneezing or coughing [1]. Usually it is caused by weak or damaged muscles and connective tissues in the pelvic floor, compromising urethral support, or by weakness of the urethral sphincter itself [2,3]. Among the main risk factors are age, pregnancy, childbirth, obesity and poor collagen turnover [4]. Typically, the first-line treatment is conservative, which includes pelvic floor training, electrical stimulation and biofeedback. In addition, Duloxetine has been licensed for treatment of SUI in women and has been shown to improve quality of life [5] but it is unclear whether the benefit is lasting [6]. If the condition does not improve, surgical alternatives can be offered. These include retropubic colposuspension, slings and urethral bulking injections [7]. Of these types of operations, the midurethral sling is currently the most common procedure performed either alone or along with concomitant pelvic floor or abdominal surgery. Transobturator tape (TOT) is similar to the initial tension free vaginal tape (TVT), but a different technique is used to insert the tape [8] However, TVT and TOT showed no significant difference concerning to the cure rate. The procedures may be comparable in terms of patient satisfaction after more than 24 month of follow-up [9,10]. The transobturator technique has the potential to reduce the incidence of significant complications associated with the retropubic approach. This technique has been further refined by the availability of the Monarc Subfascial Hammock (American Medical Systems, Inc., Minnetonka, MN, USA). Also obesity represents a risk factor for SUI [11] today there are only limited data available concerning safety, efficacy and outcome of TOT sling procedure in obese females. The aim of our study was to evaluate the clinical outcome after TOT procedure with special interest concerning different weight levels of women. A comparison of efficacy and safety of TOT between overweight and normal weight patients was performed.

## Methods

### Patient data

All consecutive women who underwent a TOT procedure at the University hospital Marburg, Department of Urology and Pediatric Urology, between January 2004 and January 2011 were retrospectively assessed (patients history, level of SUI, age, body weight etc.). They all had undergone conservative therapy options over minimal three months including urotherapy (meaning behavioural training) and pelvic floor workout*.* All patients signed an informed consent for the collection and analysis of their data.

The patients were classified into 3 groups according to the WHO body mass index (BMI): Group A (normal BMI <25kg/m^2^), Group B (overweight BMI 25-30kg/m^2^), Group C (obese BMI > 30kg/m^2^).

### Preoperative diagnostics

All patients were urodynamically diagnosed with SUI. If mixed symptoms were present, like urgency, frequency or nocturia, the stress urinary incontinence predominated on review of the patient’s history. Patients with mixed or isolated urge incontinence were excluded. Multichannel urodynamic testing included post void residual urine (PVR) determination, multichannel cystometrogram (CMG), and uroflowmetry. Patients were excluded if PVR exceeded 100 ml or CMG had detrusor overactivity. Mixed incontinence was defined by the presence of sensory urge incontinence or detrusor overactivity as well as stress incontinence during urodynamic examination.

### Surgical technique

TOT was performed using the “Monarc Subfascial Hammock” (American Medical Systems, USA). The Monarc® system includes a specially designed helical needle, which uses a transobturator-to-vagina (outside-in technique) approach for mesh placement. The procedures were performed with the patient in high lithotomy position and under spinal or general anesthesia, according to patient’s preference. All received single-shot broad-spectrum intravenous antibiotics. Patients were instructed to avoid heavy lifting, exercise, and sexual intercourse for a minimum of 4 weeks postoperatively.

### Postoperative evaluation

Intraoperative events including blood loss, time for Monarc® implantation, any complications, and additional procedures were recorded. Subjects were seen at routinely scheduled 12 and 52 weeks postoperatively. Some patients took also different dates for personal reasons. Information regarding continence status was obtained and recorded. Standardized incontinence and quality of life questionnaires as the ICI Questionnaire Short Form (ICIQ-SF) and the King’s Health questionnaire (KHQ) were used. Repeat urodynamics were not utilized. Cure was defined as no leakage (dry), or minimal leakage not requiring protection (substantially dry), reported by the patient, along with no leakage seen during an in office stress test. Also improvement was defined as a reduction in the use of pads of about 50% or more. Outcome measures reported include continence status, pad use, urinary urgency, need for medication for urgency, urinary retention (PVR >100 ml), and any healing difficulties. All terminology was in accordance with current ICS terminology, except where noted differently [12].

### Statistics

Patient demographics, clinical history, preoperative, surgical, and postoperative data were summarized using descriptive statistics for continuous variables and frequency tables for categorical variables (SPSS for Windows®, Version 17.0).

## Results

### General data

A total of 116 sequential female patients with symptomatic SUI underwent a TOT procedure at the University hospital Marburg, Deptartment of Urology and Pediatric Urology between January 2004 and January 2011. Mean age was 62.5 years (range 36–83).

Concomitant surgery with anterior or/and posterior colporrhaphy were preformed during TOT procedure in 19 women (anterior = 16, posterior =1, both = 2). Additionally in 1 patient a simultaneous resection of a urethral polyp was performed.

The range of BMI in the patients was 18–47 kg/m^2^ with a median BMI of 27.5 kg/m^2^. The patients were followed up between 2 and 56 months with a median follow up of 21 months.

### BMI-Groups

Dividing the women concerning their body weight there were 31 women in Group A (normal BMI <25kg/m^2^), 56 women in Group B (overweight BMI 25-30kg/m^2^) and 29 women in Group C (obese BMI >30kg/m^2^) with a mean age of 62 years (37–80) in Group A, 63 years (36–83) in Group B and 60 years (38–78) in Group C. The median BMI was 23,4 kg/m^2^ (18–24.8) in Group A, 28,1 kg/m^2^ (25,1-30) in Group B and 35,4 kg/m^2^ (30.1-47.2) in Group C. 25% of the patients in Group A underwent a co-therapy, 20% in Group B but only 7% in Group C.

### Operation time

Overall operation time including the cases with concomitant surgery was 40 min (10–120). No significant differences were detectable between the groups: 38.5 min (18–85) in Group A, 43.6 min (10–120) in Group B and 36 min (15–120) in Group C.

### Success of procedure

**Figure 1:**
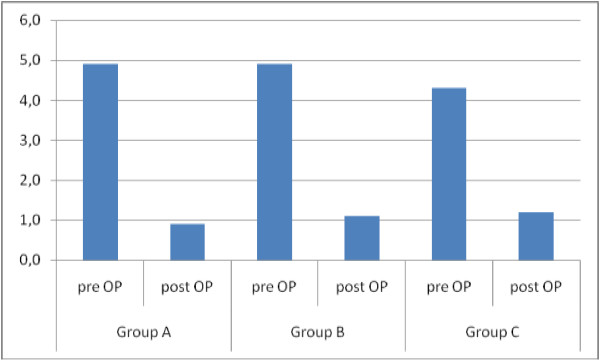
Pad consumption before and after surgery broken down by groups.

After TOT-procedure the used median pads/day significantly reduced from baseline 4 (1–16) to 0 (0–9) (p < 0.01), without showing some significant differences between the BMI-groups. We found a significant improvement after surgery without significant differences between the groups (Figure 1).

Used pads/day after 6, 12, 24 and 36 months showed a significant reduction compared to baseline in every group without significant differences between the respective BMI-groups. Comparing possible impairment during time period (6, 12, 24, 36 months) no significant increase of pads/day were evident in every group and between Group A, Group B and Group C (Table 1)*.*

**Table 1:** Median used pads/day 6, 12, 24 and 36 months after TOT-procedure in group A, B and C (n = number of patients, range of used pads)

	**6 months**	**12 months**	**24 months**	**36 months**
**Group A**	1 (n = 30, 0-4)	1 (n = 28, 0-4)	1 (n = 18, 0-4)	0 (n = 11, 0-1)
**Group B**	0 (n = 52, 0-9)	0 (n = 35, 0-6)	1 (n = 23, 0-6)	0 (n = 10, 0-3)
**Group C**	0 (n = 25, 0-7)	0.5 (n = 21, 0-7)	0.5 (n = 16, 0-2)	0 (n = 10, 0-1)

Success rate was 93% with a cure rate of 83% and improvement in 10% of the women. No significant differences were evident between the groups (Group A: 96%, Group B: 94%, Group C: 91%).

Logistic analysis also revealed that BMI, history of previous pelvic surgery, concomitantly performed pelvic surgery, or occurrence of complications did not affect cure rates.

### Complications

Overall complication rate was 23.2% (27/116) including 1 major (bleeding) and 26 minor complications (see Table 2). Postoperative temporary voiding difficulty was the main postoperative complication (n = 21, 18.1%). We found appearance of de-novo-urge in 15 women (12.9%) and temporary obstructive voiding in 6 women (5.2%) with a urinary retention in 2 women (1.7%). Mean residual urine volume increased to 75 ml, only 3 patients showed an increase above 100 ml (up to 330 ml). Voiding was not impaired by reduction of the flow time. There was no significant change after performing the operation while the range about the flow was 5–57 sec. Also the maximum flow was not significantly affected.

**Table 2:** Postoperative complication rate was 23.2% including 1 major and 26 minor complications

	**Bleeding**	**De-novo-urge**	**Dyspareunia/ vaginal pain**	**Residual urine**	**Vaginal erosion**
**Group A**	1	4	1	3	1
**Group B**	Ø	7	3	2	Ø
**Group C**	Ø	4	Ø	1	Ø

Complication rates between the BMI-groups were nearly similar without significant differences.

### Patients satisfaction

**Figure 2:**
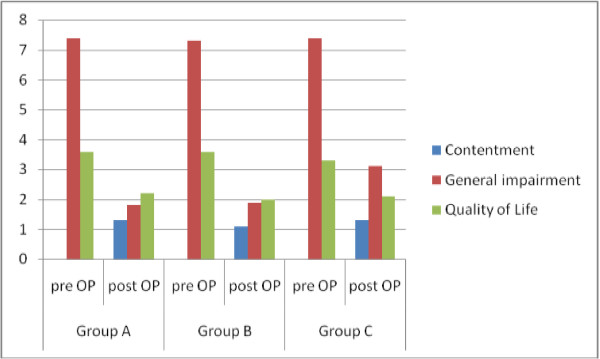
KHQ showed significant improvement in QoL without differences between the three groups.

KHQ showed significant improvement in Quality of Life in all circumstances for all different groups. No significant differences between the groups were notable. Majority of women (93%) are satisfied with the TOT-procedure including individual outcome and recommend the procedure, also without significant differences between the groups (Group A: 100%, Group B: 90%, *Group C*: 89.5%). We used the question “Would you repeat this procedure?” representative of contentment, so there could only be a measurement in the postoperative evaluation (Figure 2).

All relevant data, divided into different BMI-groups were summarized in Table 3.

**Table 3:** Summarized data (divided into different BMI-groups)

	**No**	**Age (years)**	**Median BMI (kg/m**^**2**^**)**	**Op-time (min)**	**Compl. (n)**	**Success rate**	**Pads (n/day)**	
							**Pre**	**Post**
**Group A (-25 kg/m**^**2**^**)**	31	62.2 (37-80)	23.4 (18-24.8)	38.5 (18-85)	10	96%	4.5 (1-11)	0.85 (0-4)
**Group B (25-30kg/m**^**2**^**)**	56	63 (36-83)	28.1 (25.1-30)	43.6 (10-120)	12	94%	4.7 (1-16)	1.1 (0-9)
**Group C (>30kg/m**^**2**^**)**	29	60.3 (38-78)	35.4 (30.1-47.2)	36 (15-120)	5	91%	5 (3-7)	1.4 (0-6)
**Σ**	116	**62** (36-83)	**27.5** (18-47.2)	**40** (10-120)	**27** (25.5**%**)	**93%**	**4** (1-16)	**0** (0-9)

## Discussion

This study showed that TOT was equally safe and effective for treating SUI regardless of BMI. The postoperative quality of life was similarly improved for women in each weight groups, and there were no significant differences in the complication rate.

BMI is significantly correlated with intra-abdominal pressure [13] which may increase stress on the pelvic floor, contributing to the development and recurrence of SUI. However, we did not find that women with a higher BMI were more likely to have SUI on follow-up. Results of the studies of incontinence surgery in obese patients have been contradictory. Responses to a mailed questionnaire by 970 women who underwent TVT indicated a markedly unfavorable outcome in 61 very obese women (BMI > 35 kg/m^2^) [14]. The overall cure rate in 291 women of normal weight was 81.2%, as compared to 52.1% in the 61 very obese. In other studies these data could not be confirmed and reasons for this poor outcome are not clear. Maybe it is due to the limitations of a mailed questionnaire reflecting the general enhanced dissatisfaction of obese people [9,15]. Another study evaluated 195 women who underwent TVT. At one year of follow-up, they found a similar surgical outcome among 68 normal-weighted, 65 overweight and 62 obese women [16]. The proportion of subjects with SUI one year after surgery was 18% in the obese, 14% in the overweight, and 19% in the normal weight groups, differences were statistically not significant. Skriapas et al. compared 31 morbidly obese patients with BMI > 40 kg/m^2^ and 52 patients with a BMI of <30 kg/m^2^ with a mean follow-up of 18.5 months. The objective cure rate in control group was 92.3% and 86.9% for morbidly obese group, so no significant difference was shown. They thus suggested that TVT is a good option for morbidly obese patients with severe stress incontinence [17]. Differences between the studies are caused by different lengths of follow-up, variation in the type of continence surgery, and different definitions of cure. Therefore comparability between this studies is limited (may not always be given). Also in two studies using the transobturator tape procedures no significant association between BMI and surgical outcome were found [18,19]. Even if cure was defined differently in these two studies the results are in line with our findings after a follow up of nearly 2 years. In the publication of Po-En Liu and coworkers the gain was the use of objective as well as subjective measures of cure. Compared with the normal weight group, women with a higher BMI did appear to have a lower objective cure rate. Objective cure was defined as no urine leakage during the stress test in the filling phase of urodynamic studies. The statistical analysis did not indicate any significant differences among the groups, although it is possible that some difference might be found if the number of cases were higher. Instead of the ICIQ-SF and KHQ they used UDI-6 and IIQ-7 scores. However, they also find a significant improvement in all compared groups so that the TOT procedure indeed improves quality of life regardless of BMI.

Liapis et al. reported an objective cure in 82.4% of 115 subjects after TOT based on the pad test finding at 4 years postoperatively [20], while there was a slightly lower objective success rate at a median of 24 months of follow-up. Albo et al. described similar objective success rates comparing TVT (77.3%) and TOT (72.3%) after 24 month follow-up in 516 women [10]. In our population success rate of 93% after 21 month of follow up was similar with cure rate of 83% and improvement of 10%.

In 2012 also a one-year outcome of mid-urethral sling procedures for stress urinary incontinence according to body mass index was published. The retrospective clinical trial was performed with 284 patients treated by the SPARC sling procedure and 49 patients also treated by the MONARC sling procedure. The women were also classified into 3 groups by BMI according to the WHO Expert Consultation. Patient’s characteristics and clinical outcomes of the operation were analyzed according to BMI at 1 year after surgery but only via questionnaires and interviews. The objective and subjective cure rates for the obese group were worse after TOT procedure (96.8% vs. 66.7%) but because of the small sample size of the TOT group not statistically significant [21]*.* A limitation of our investigation was the duration of follow up. However, there are no publications comprising a follow up of 5 or more years also performing a differentiated approach on the outcome of obese female. So are the outcomes of transobturator tape procedure generally durable in long-term follow-up? A 5-year follow-up study comparing Burch colposuspension and transobturator tape for the surgical treatment of SUI in person without another concomitant procedure showed, that the 5-year cure rates were similar (objective cure rate, 73.9% versus 77.5%; subjective cure rate, 76.8% versus 81.7% [22]. Yonguc et al. showed in their publication that objective cure, subjective cure and patient satisfaction rates of the 126 women at 1 year after transobturator tape procedure were 89.6, 86.5 and 92% respectively. During 5-year follow-up, objective cure rate was stable with 87.3% rate, whereas subjective cure and patient satisfaction rates were decreased to 65.9 and 73%, respectively [23]. The group of Heinonen et al. evaluated 191 patients underwent TOT procedure, and thereby 139 (73%) after a mean follow-up of 6.5 years. The patient cohort was heterogeneous and consisted of patients with SUI and mixed urinary incontinence (MUI) as well as recurrent SUI. There objective and subjective cure rates were 89% and 83%, respectively. Subjective cure rate was 84% at 20-month follow-up and was maintained up to 6.5 years. At long-term follow-up, the objective cure rate was slightly greater than the subjective cure rate. In their evaluated cohort, mean body mass index BMI was 28 (range, 19–45). Patients with BMI higher 30 had significantly higher scores on condition-specific questionnaires and significantly lower scores on quality of Life questionnaires, indicating a lower general quality of life and health than in patients with BMI less or equal 30 [24]. In the subgroup analysis of our sample we could not confirm this effect for the objective cure rate.

Another limitation of our investigation was the sample size. We deliberately limited our sample to women with isolated SUI. On the other hand, our population was representative of women typically seen in the clinic complaining of isolated SUI. Our results thus help confirm the effectiveness of the TOT procedure for women in a general clinical setting.

## Conclusions

Our data demonstrate that TOT procedure is safe and effective. BMI did not influence the outcome of TOT procedures at a median of 21 months after surgery and represents no contraindication for continence surgery.

### Ethical/Institutional review board approval

There was no IRB Approval acquired for this study. An approved medical product was used. Data collections as well as pre- and postoperative investigations were performed within the usual and guideline based visits.

## Competing interest

The authors declare that they have no competing interest.

## Authors’ contributions

CF: Project development, Data collection, Data analysis, Manuscript writing. FL: Data collection. ZV: Data collection, Manuscript editing. PJO: Manuscript editing. RH: Manuscript editing. AH: Project development, Data collection, Data analysis, Manuscript writing. All authors read and approved the final manuscript.

## Pre-publication history

The pre-publication history for this paper can be accessed here:

http://www.biomedcentral.com/1471-2490/14/20/prepub
